# Quantifying the spatial spillover effects of non-pharmaceutical interventions on pandemic risk

**DOI:** 10.1186/s12942-023-00335-6

**Published:** 2023-06-07

**Authors:** Keli Wang, Xiaoyi Han, Lei Dong, Xiao-Jian Chen, Gezhi Xiu, Mei-po Kwan, Yu Liu

**Affiliations:** 1 Institute of Remote Sensing and Geographical Information Systems, School of Earth and Space Sciences, Peking University, Beijing, China; 2 Beijing Key Lab of Spatial Information Integration & Its Applications, Peking University, Beijing, 100091 China; 3 The Wang Yanan Institute for Studies in Economics (WISE), Xiamen University, Xiamen, 361005 China; 4 School of Economics, Xiamen University, Xiamen, 361005 China; 5 Institute of Space and Earth Information Science, The Chinese University of Hong Kong, Hong Kong, China; 6 Department of Geography and Resource Management, The Chinese University of Hong Kong, Hong Kong, China

**Keywords:** COVID-19, Non-pharmaceutical interventions, Spatial spillover effects, SEIR, Human mobility

## Abstract

**Background:**

Non-pharmaceutical interventions (NPIs) implemented in one place can affect neighboring regions by influencing people’s behavior. However, existing epidemic models for NPIs evaluation rarely consider such spatial spillover effects, which may lead to a biased assessment of policy effects.

**Methods:**

Using the US state-level mobility and policy data from January 6 to August 2, 2020, we develop a quantitative framework that includes both a panel spatial econometric model and an S-SEIR (Spillover-Susceptible-Exposed-Infected-Recovered) model to quantify the spatial spillover effects of NPIs on human mobility and COVID-19 transmission.

**Results:**

The spatial spillover effects of NPIs explain $61.2\%$ [$95\%$ credible interval: 52.8-$84.4\%$] of national cumulative confirmed cases, suggesting that the presence of the spillover effect significantly enhances the NPI influence. Simulations based on the S-SEIR model further show that increasing interventions in only a few states with larger intrastate human mobility intensity significantly reduce the cases nationwide. These region-based interventions also can carry over to interstate lockdowns.

**Conclusions:**

Our study provides a framework for evaluating and comparing the effectiveness of different intervention strategies conditional on NPI spillovers, and calls for collaboration from different regions.

**Supplementary Information:**

The online version contains supplementary material available at 10.1186/s12942-023-00335-6.

## Background

Pandemic transmission and policy interventions are interdependent among regions. Interventions to contain the virus spread may go beyond the regions it directly affects [1, 2]. Specifically, interventions of one region may exert spatial spillover effects (SSE hereafter) to neighboring regions by affecting the human behavior of nearby regions. For instance, shelter-in-place orders in one region can restrict intraregional human mobility, leading to a decreased population flow in regions without shelter-in-place orders [3]. Ignoring such SSE may result in a biased assessment when evaluating the effectiveness of interventions.

Governments usually enact a series of non-pharmaceutical interventions (NPIs) – such as social distancing, school closures, and even national lockdowns – to combat infectious disease [4]. The COVID-19 pandemic serves as a prime example, with numerous studies exploring the necessity for NPIs and the impact of interventions on COVID-19 transmission [5–12]. The most common method used in these studies is the infectious disease dynamics model. These models divide populations or other hosts into different compartments based on their health status (for example, susceptible, infected, exposed, etc.), and then develop transformation rules based on disease transmission characteristics to simulate the transmission dynamics of infectious diseases [13]. Therefore, they are also called compartmental models, which mainly include SIR (Susceptible - Infected - Removed) and SEIR (Susceptible - Exposed - Infected - Removed). They can simulate the spread of infectious diseases over time and space as well as the influence of interventions on transmission dynamics. Researchers can simulate the spread of infectious diseases over time and space, as well as the effect of interventions on the transmission process. Scholars can add compartments [14] or set different contact or mobility characteristics for populations to evaluate interventions [15], or adjust inter-regional human flow to simulate the effects of inter-regional lockdown [9, 16]. Yet, most of these studies operate under the assumption that interventions only have an impact within the region where they are implemented [17, 18], disregarding any spillover effects across regions. The SSE of NPIs and the effectiveness of different intervention strategies conditional on SSE are largely unknown.

The spillover effect occurs when the impact of policies or factors in one region extends beyond its borders, affecting neighboring regions. To assess the spillover effects, several methods have been developed and used in fields such as crime [19], air pollution [20], human mobility [21, 22], and infectious diseases [23, 24]. With regard to COVID-19, several studies have acknowledged the presence of spillover effects. The first direction focuses on the spatial spread of COVID-19 by studying the relationship between cases in different regions [25, 26], suggesting that factors such as deaths, recoveries, or vulnerability in one region could affect confirmed cases in nearby regions. The second direction focuses on analyzing the disease’s spread over time and space, demonstrating that there is a high degree of spillover among regions [27]. The third direction focuses on the role of interventions in spillover effects related to COVID-19. These researches suggest that interventions can extend their reach, leading to better policy outcomes [28]. Furthermore, studies examining specific policies such as social distancing or place closures have highlighted the significant spillover effects of these policies in areas with both geographic and social network proximity, and considerable differences in the spillover effects produced by various types of places [1, 2, 29]. However, existing studies tend to focus on identifying SSE and exploring the causal relationship between policies or environmental factors and cases or human movement, using various econometric methods. Comprehensive analyses, especially studies that combine compartmental mathematical model and counterfactual simulations to evaluate and compare the effectiveness of different intervention strategies conditional on SSE, are very limited.

**Fig. 1:**
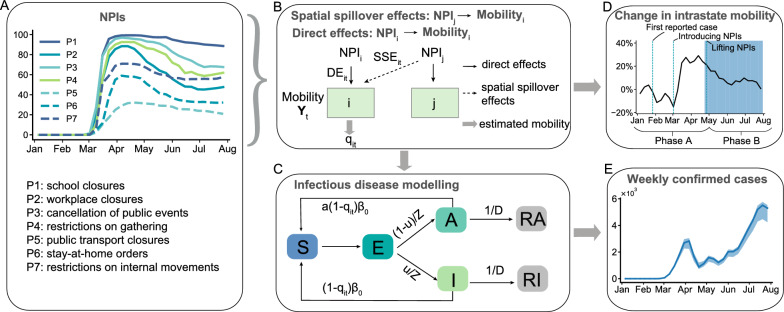
The modeling framework of SSE. **A** The average intensity of different NPIs for all regions by week. **B** The influence of NPIs on intrastate human mobility from the spatial panel model. The mobility in region i can be influenced by the NPIs in the regions *i* and *j*. Therefore, the changes in intrastate human movement $Y_t$ can be explained by the sum of direct effects and SSE of NPIs and other effects. We use $q_{it}$ to represent the estimated changes in intrastate human movement from the spatial panel model. **C** The transmission among different states of individuals. *S*, *E*, *I*, and *A* indicate susceptible, exposed, symptomatic, and asymptomatic individuals, respectively. *RI* and *RA* represent the removed symptomatic and asymptomatic individuals. $(1-q_{it})\beta _0$ indicates the transmission rate of symptomatic infected persons influenced by NPIs. *a* is the transmission rate of asymptomatic infected persons relative to symptomatic infected persons. *Z* and *D* denote the mean incubation period and infection period, whose inverse stand for the probability that exposed individuals become symptomatic infected individuals or that infected individuals are removed, respectively. $\mu$ is the proportion of symptomatic patients among all infectious patients. **D** The average intrastate human mobility trend across regions. **E** The estimated weekly confirmed cases of the Washington State

With the aim of filling the aforementioned gap, we develop a quantitative framework utilizing US state-level policy data and mobile phone data. The framework includes a panel spatial econometric model and an S-SEIR model (Spillover-Susceptible-Exposed-Infected-Recovered), to evaluate the SSE of NPIs on COVID-19 (Fig. 1). To avoid confusion with the states in the epidemic model, the term region is used below to denote the US state. We assume that the implementation of NPIs would affect disease transmission by influencing human mobility. To quantify the relationship between NPIs and human mobility, we use the panel spatial econometric model to isolate the SSE ($NPI_j \rightarrow mobility_i$, where *i* and *j* represent regions *i* and *j*) and direct effects ($NPI_i \rightarrow mobility_i$) of NPIs (Fig. 1B). Then, we treat the estimated change of intrastate human mobility from the panel spatial econometric model as input parameters to adjust the transmission rate in the S-SEIR model, and simulate the SSE and direct effects of NPIs on COVID-19 cases using the S-SEIR model. We further consider different scenarios that vary by the intervention regions and interstate flows. The simulation results highlight the importance of intervening in regions with high human mobility in reducing the national cumulative confirmed cases. Our findings also show that lockdowns in targeted regions with high human mobility can effectively reduce more cases. This research proposes a quantitative framework to isolate the direct effects and SSE of different NPIs on human mobility and infected cases. The framework can help one understand fully the scope of policy influence to coordinate regional resources and search for more effective interventions such as region-based policies to fight against pandemic risk.

## Study area and data

This study focuses on evaluating the impact of restricting social distance on human mobility and COVID-19 transmissions in the United States. We focus on the state level, as most NPIs policies are implemented on such spatial resolution. Our research covers the continental United States includes 48 states and Washington D.C., given the policy and human mobility data availability. To this end, three datasets are introduced, with the first one being the case data. This data is obtained from the New York Times [30] and provides the confirmed cases and death statistics at the state level in the United States. The first case of COVID-19 in the United States was reported on January 20th  [31], and since then, the country has gone through several epidemic waves. This study, however, focuses on the first wave of the outbreak in the US, which occurred from January to August 2020.

The policy data are obtained from the Oxford COVID-19 Government Response Tracker (OxCGRT) [32]. The OxCGRT keeps track of US state-level policy responses since January 1, 2020, shown in the time-series format, and provides ordinal policy intensity and records geographic scope [32]. We focus on seven human mobility-related NPIs ($P_1-P_7$) (Fig. 1A), specifically, including school closures, workplace closures, cancellation of public events, restrictions on gatherings, public transport closures, stay-at-home orders, and restrictions on internal movements. To compare the intensity of these interventions across different regions, we calculated the intensity of each policy index and aggregated them into weekly intervention intensity scores (Table  1). The intensity score ranges from 0 to 100. Based on the timing of the interventions, we define two phases based on the reopening time of NPIs: Phase A (January 6, 2020 - April 26, 2020), an early phase in which interventions were introduced; and Phase B (April 27, 2020 - August 2, 2020), a lifting phase in which interventions were relaxed [33, 34].

**Table 1:** Summary of meaning and stringency of different NPIs in US states

Variables	Description	Mean	Std. Dev.	Min	Max
School closures	Record closings of schools and universities	63.39	44.50	0	100
Workplace closures	Record closings of workplaces	42.75	34.64	0	100
Cancellation of public events	Record cancelling public events	56.54	42.72	0	100
Restrictions on gatherings	Record limits on gatherings	50.29	43.61	0	100
Public transport closures	Record closing of public transport	17.24	25.44	0	100
Stay-at-home orders	Record orders to “shelter-in-place” and otherwise confine to the home	27.32	24.01	0	66.7
Restrictions on internal movement	Record restrictions on internal movement between cities/regions	40.73	33.89	0	100

During the COVID-19 pandemic, various data sources emerged to characterize human movement, including SafeGraph, Google, Apple, Descartes Labs, and Facebook. Kang et al. compared these sources and generated a dynamic human mobility flow dataset for the United States based on data from SafeGraph [35]. They analyzed the visits of millions of anonymous mobile phone users to various locations, computed daily and weekly origin-to-destination (OD) human flow data, and aggregated the data at three geographic levels: census tract, county, and state. In this study, we select state-level daily human mobility data and aggregate it to the weekly level. The change of weekly intrastate movement $Y_{it}$ of the region *i* at week *t* is:

1 $$\begin{aligned} Y_{it} = \frac{flow_i(0)-flow_{i}(t)}{flow_{i}(0)}, \end{aligned}$$

where $flow_{i}(0)$ is the average human mobility in region *i* at the early stage without being affected by NPIs (from January 6, 2020, to February 2, 2020, four weeks), and $flow_i(t)$ denotes the human mobility in region *i* at week *t*. $Y_{it}$ greater than 0 indicates that intrastate human movement at time *t* is lower than the early stage without being affected by NPIs, otherwise indicates that human movement at time *t* is higher than that period. Fig. 1D illustrates the change in intrastate human mobility. Prior to the implementation of the interventions, intrastate human mobility is at a high level, but decreases sharply after the NPIs are implemented. With weakened interventions, human mobility slowly picks up.

## Methods

### A spatial panel model for NPIs and human mobility

To study the interplay between NPIs and mobility, we employ the panel spatial autoregressive (SAR) model to obtain direct effects and SSE from different NPIs [36]. Unlike the linear panel model, the spatial panel model considers the dependence between geographically adjacent units [37]. This enables us to capture the SSE across geographical units [25].

2 $$\begin{aligned} \varvec{Y_t} = \rho \varvec{W Y_t} + \varvec{ X_{t-1}} \varvec{\lambda } + \varvec{\nu } +l_N \xi _t + \varvec{\epsilon _{t}}, \end{aligned}$$

where ${\varvec{Y}}_{{\varvec{t}}}$ denotes the change of human mobility for 49 regions at week *t*. ${\varvec{X}}_{{\varvec{t-1}}}$ contains in total seven 1-week lagged NPI intensities for 49 regions, and $\varvec{\lambda }$ is the corresponding slope coefficient. $\varvec{W}$ denotes the spatial weights matrix that captures the connectivity of 49 regions. $\varvec{W Y_t}$ represents the spatial lag term of intrastate human mobility and $\rho$ denotes the spatial effect across regions. $l_N$ represents a $49 \times 1$ column vector of ones. $\nu$ and $\xi _t$ are, respectively, $49 \times 1$ vector of regional effects and a scalar time effect. $\epsilon _t = (\epsilon _{1t},\cdots , \epsilon _{nt})^T$ is a vector of disturbance terms, where $\epsilon _{it}$ is assumed to be independently and identically normally distributed for all *i* with zero mean and variance $\sigma ^2$.

Note that the spatial weight matrix is an important pre-defined input for the spatial econometric model. The spatial weight matrix $\varvec{W}=[W_{ij}]$ reflects the degree of adjacency or association between spatial units that can be specified based on the adjacency or distance relationship [38]. However, it’s important to keep in mind that the transmission of infectious diseases, such as COVID-19, may not always be limited to geographically close regions. Geographical proximity does not fully capture air-traffic-mediated epidemics [39]. Thus, determining neighbors merely from geographical distance is not sufficient. To overcome this limitation, we use the spatial interaction distance, which captures the dependence relationship by human interaction across regions. Following the concept of KNN, we construct the K maximum interaction matrix (KMI) based on the spatial interaction distance. Like KNN, the key aspect of the KMI method is selecting the K-value and calculating the distances. We define the distance by origin–destination (OD) flows between region *i* and *j* as: $d(i,j) = flow_{ij}$, if $i = j$, $d(i,i) = 0$. Then, we identify the first *k* regions with the closest interaction distances to region *i* as its neighbors. To determine the value of *k*, we use the deviance information criterion (DIC) as described in Gelman [40]. The KMI is constructed based on the interstate human mobility data from 6 January to 2 February 2020 and all spatial weight matrices are normalized. The detailed specifications of the spatial weights can be found in the Supplementary materials.

### Derivation of direct and spatial spillover effects of NPIs

Following [36, 41], we rely on the following reduced form of Eq. 2 to derive the direct effects and SSE:

3 $$\begin{aligned} \begin{aligned} \varvec{Y_t}&= (\varvec{I_n} -\rho \varvec{W})^{-1} \varvec{X_{t-1} \lambda }+ (\varvec{I_n} -\rho \varvec{W})^{-1}(\varvec{\nu } + l_N \xi _t + \varvec{\epsilon _t}), \end{aligned} \end{aligned}$$

where $\varvec{I_n}$ is the identity matrix of dimension *n*. The $n \times m$ matrix of $\varvec{X_{t-1}}$ can be partitioned by columns as $\varvec{X_{t-1}}=(X_{1,t-1},\cdots ,X_{m,t-1})$, where $X_{r,t-1}=(x_{r1,t-1},x_{r2,t-1},\cdots ,x_{rn,t-1})'$ is the $n \times 1$ vector of the *r*th NPI measure for all *n* regions and $x_{ri,t-1}$ represents the *r*th NPI measure of region *i* at period $t-1$. Denote $V(\varvec{W})=(\varvec{I_n} -\rho \varvec{W})^{-1}$ and $U_r(\varvec{W} )=V(\varvec{W}) \lambda _r$, with $\lambda _r$ being the *r*th coefficient of $\varvec{\lambda }$. Equation 3 can be rewritten as:

4 $$\begin{aligned} \varvec{Y_t}&=V(\varvec{W}) \sum _{r=1}^m \lambda _r X_{r,t-1}+ V(\varvec{W}) (\varvec{\nu } + l_N \xi _t ) +V(\varvec{W}) \varvec{\epsilon _t}, \nonumber \\&=\sum _{r=1}^m U_r(\varvec{W})X_{r,t-1}+ V(\varvec{W}) (\varvec{\nu } + l_N \xi _t ) +V(\varvec{W}) \varvec{\epsilon _t}. \end{aligned}$$

Let $U_{rij}(\varvec{W})$ be the $i\hbox {th}$ row and $j\hbox {th}$ column element of $U_r(\varvec{W})$, and $V(\varvec{W})_i$ be the $i\hbox {th}$ row of $V (\varvec{W})$. The $i\hbox {th}$ row of Eq. 3 reads:

5 $$\begin{aligned} \begin{aligned} Y_{it}=\sum _{r=1}^m [U_{ri1}(\varvec{W})x_{r1,t-1}+\cdots +U_{rin}(\varvec{W})x_{rn,t-1}] + V(\varvec{W})_i (\varvec{\nu } + l_N \xi _t ) +V(\varvec{W})_i \varvec{\epsilon _t}. \end{aligned} \end{aligned}$$

Following LeSage and Pace [36], the marginal effect of the *r*th NPI measure of region *j*, namely, $x_{rj,t-1}$ on the intrastate human mobility of region *i*, $Y_{it}$ is

6 $$\begin{aligned} \frac{\partial Y_{it}}{\partial x_{rj,t-1}}=U_{rij} (\varvec{W}), \end{aligned}$$

for $j=1,2,\cdots ,n$. In particular, the direct effect from region *i*’s own NPI can be captured by $U_{rii} (\varvec{W})$, the *i*th diagonal element of $U_{r} (\varvec{W})$. Hence, we can derive the overall direct effect and SSE of all NPI measures on $Y_{it}$, by adding the direct effect of all NPIs from region *i*, as well as the NPI spillovers from all other regions:

7 $$\begin{aligned} \begin{aligned} DE_{it}&= \sum _{r=1}^{m} U_{rii} (\varvec{W}) x_{ri,t-1}, \\ SSE_{it}&= \sum _{r=1}^{m} \sum _{j=1, j \ne i}^{n} U_{rij} (\varvec{W}) x_{rj,t-1}. \end{aligned} \end{aligned}$$

The intrastate human mobility of region *i* can then be expressed as:

8 $$\begin{aligned} \begin{aligned} Y_{it} = DE_{it} + SSE_{it} + V(\varvec{W})_i (\varvec{\nu } + l_N \xi _t ) +V(\varvec{W})_i \varvec{\epsilon _t}, \end{aligned} \end{aligned}$$

which further implies

9 $$\begin{aligned} E(Y_{it})=DE_{it} + SSE_{it} + V(\varvec{W})_i (\varvec{\nu } + l_N \xi _t ). \end{aligned}$$

In terms of estimation, we use the summation of the posterior means of the overall direct effect, the SSE, and the regional and time effect influence $V(\varvec{W})_i (\varvec{\nu } + l_N \xi _t )$ to approximate $Y_{it}$. Additionally, we set the estimated value of $Y_{it}$ equal to $q_{it}$, which means $q_{it}$ is the estimated change of intrastate human mobility. We use the Bayesian Markov Chain Monte Carlo (MCMC) method to estimate the coefficients of panel spatial econometric model. Details on the MCMC estimation algorithm as well as the direct effect and SSE estimates are provided in the Supplementary materials.

Based on the spatial panel SAR model, we evaluate the impact of NPIs on intrastate human mobility. The change in human mobility can significantly affect the transmission of infectious diseases like COVID-19. For example, the stay-at-home order restricts people’s travel and reduces human flow in the region, which minimizes contact between infected and susceptible individuals and ultimately contains infectious disease transmission. Therefore, many studies use the change in human mobility before and after interventions to construct infectious disease models that simulate disease transmission and evaluate the impact of policies [42, 43]. Here we use the estimated changes ($q_{it}$) in human movement to develop the infectious disease model because it includes the direct effects and SSE of NPIs, which can help us simulate the effect of SSE of NPIs on disease transmission.

### Parameter inference of S-SEIR

The introduction of the classical SEIR model is necessary prior to develop our infectious disease model, as it serves as an important reference. The basic form of the SEIR model can be expressed as:

10 $$\begin{aligned} \begin{aligned} \frac{\textrm{d}S}{\textrm{d}t} =&-\beta \frac{I S}{N} \\ \frac{\textrm{d}E}{\textrm{d}t} =&\beta \frac{I S}{N} -\frac{E}{Z} \\ \frac{\textrm{d}I}{\textrm{d}t} =&\frac{E}{Z} - \frac{I}{D}, \\ \frac{\textrm{d}R}{\textrm{d}t} =&\frac{I}{D}, \\ \end{aligned} \end{aligned}$$

where *S*, *E*, *I*, and *R* are susceptible, exposed, infected, and removed populations, *N* denotes the total population ($N = S + E + I + R$). $\beta$ denotes the transmission rate, which is related to disease characteristics and population exposure. *Z* and *D* denote the incubation and infection periods. The inverse of the incubation period indicates the fraction of exposed individuals that become infected, while the inverse of the infection period indicates the fraction of infected individuals who recover or dead. Classical SEIR models typically assume that many disease characteristics, such as the transmission rate and infection period, are constant. However, this assumption does not apply to the complex transmission of COVID-19.

The change of intrastate human mobility can reflect the influence of NPIs [44], which may also affect the transmission rate of infectious diseases. When the interventions occur, the transmission rate can be quantified as $\beta _t = (1-q) \beta _0$, with *q* being the proportion of an infectious individual’s daily susceptible contacts who will not go on to develop diseases and thus can be temporarily removed from the susceptible pool [45]. $\beta _{0}$ is the initial transmission rate. To incorporate the effects of NPIs on the transmission rate, we utilize the estimated changes ($q_{it}$) in intrastate human mobility from our panel spatial econometric model to represent the number of removed susceptible contacts. Meanwhile, the estimated changes in intrastate human mobility can help us isolate the direct effects and SSE of NPIs. Therefore, we can develop the S-SEIR metapopulation model, taking into account the role of SSE on intrastate human mobility change during disease transmission.

11 $$\begin{aligned} \begin{aligned} \frac{\textrm{d}S_i}{\textrm{d}t} =&-(1-q_{it}) \beta _{0} \frac{I_i S_i}{N_i} - a (1-q_{it}) \beta _{0} \frac{A_i S_i}{N_i} \\&+ \theta \sum _{j} \frac{\Delta M_{ji}S_j}{N_j - I_j} - \theta \sum _{j} \frac{\Delta M_{ij}S_i}{N_i - I_i}, \\ \frac{\textrm{d}E_i}{\textrm{d}t} =&(1-q_{it}) \beta _{0} \frac{I_i S_i}{N_i} + a (1-q_{it}) \beta _{0} \frac{A_i S_i}{N_i} - \frac{E_i(t)}{Z}\\&+ \theta \sum _{j} \frac{\Delta M_{ji}E_j}{N_j - I_j} - \theta \sum _{j} \frac{\Delta M_{ij}E_i}{N_i - I_i}, \\ \frac{\textrm{d}I_i}{\textrm{d}t} =&\mu _i \frac{E_i}{Z} - \frac{I_i}{D_i}, \\ \frac{\textrm{d}A_i}{\textrm{d}t} =&(1 - \mu _i) \frac{E_i}{Z} - \frac{A_i}{D_i} + \theta \sum _{j} \frac{\Delta M_{ji}A_j}{N_j - I_j} - \theta \sum _{j} \frac{\Delta M_{ij}A_i}{N_i - I_i}. \end{aligned} \end{aligned}$$

$S_i$, $E_i$, $I_i$, $A_i$, and $N_i$ are susceptible, exposed, symptomatic infected, and asymptomatic infected persons and total population of region *i*, with $S_i + E_i + I_i + A_i + RI_i + RA_i = N_i$. $RI_i$ and $RA_i$ are removed for symptomatic and asymptomatic patients, respectively. $\textrm{d}RI_i/\textrm{d}t = {I_i}/{D_i}$, $\textrm{d}RA_i / \textrm{d}t = {A_i}/{D_i}$. For model parameters, $\beta _{0}$ can be expressed as $\beta _0 = R_0 /D$, in which $R_0$ is the basic reproductive number, indicating the average number of people infected by an infectious person. $(1-q_{it}) \beta _{0}$ indicates the transmission rate of symptomatic infected individuals, adjusted by the NPI direct effects and SSE influence of region *i* at time *t*. *a* is the transmission rate of asymptomatic infected persons relative to symptomatic infected persons. $\mu _i$ denotes the reported fraction of symptomatic infections in region *i*, $D_i$ is infectious period in region *i*. In addition, our model characterizes the interactions and movements between different subpopulations across time and space [46–48]. Specifically, the human movement among regions leads to weekly changes in the population of each region: $N_i(t+1) = N_i(t) + \theta \sum _{j} \Delta M_{ji}(t) - \theta \sum _{j} \Delta M_{ij}(t)$, $\Delta M_{ij}(t)$ denotes the population flows from region *i* to *j* at week *t*. $\theta$ is an adjustment factor to adjust the human movement data as it is only a sample data set. Inter-regional population movement can cause case exchange across regions. We assume that symptomatic individuals are immobile but susceptible, exposed, and asymptomatic patients can move across regions. $\Delta M_{ij}S_i$, $\Delta M_{ij}E_i$ and $\Delta M_{ij}A_i$ denote the susceptible, exposed, asymptomatic patients moving from *i* to *j*, respectively.

**Fig. 2:**
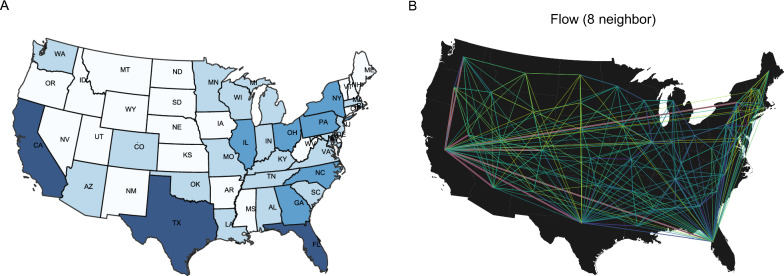
Geographical representation of class map and spatial weight matrix.**A** The class map by Kmeans method according to the intrastate human mobility. Note: US states are divided into four groups: (1) Region 1 ($r_1$): 22 regions with relatively low human mobility; (2) Region 2 ($r_2$): 18 regions with intermediate human mobility; (3) Region 3 ($r_3$): 6 regions with higher human mobility; (4) Region 4 ($r_4$): 3 regions with the highest human mobility. **B** Flow matrix with nearest 8 neighbors: $49 \times 49$ interstate interaction matrix, where each region is only connected to its closest 8 neighbors. The purple line represents neighbors of California. The color indicates the size of the element of the spatial weights. The darker the color, the larger the weight value

Following recent literature [49–53], we estimate model parameters by an iterative Bayesian inference algorithm [ensemble adjustment Kalman filter (EAKF)]. This method has been applied to metapopulation models and to infer epidemiological parameters for many infectious diseases successfully [50, 52, 54]. EAKF is a Monte Carlo implementation of the Bayesian update problem. It combines observations with the probability density function (prior) generated by the model simulation over time to generate a posterior estimate of the model state variables (including the variables and parameters). Therefore, we can estimate the four variables(*S*, *E*, *I*, *A*) of each region and infer six model parameters ($R_0$, *a*, $\theta$, $\mu$, *Z*, *D*).

The spread and evolution of infectious diseases is a dynamic process, and their associated epidemiological parameters can vary over time and region. For instance, the infection period may be influenced by factors such as hospital admission policies and contact tracing. Accelerated hospital admissions or early isolation of close contacts can significantly shorten the infection period. Meanwhile, the reporting rate of the cases can be impacted by testing and record-keeping practices, which can vary across regions. Hence, we assume that the reporting rate ($\mu _i$) and the infection period ($D_i$) are subject to change with time and region, while the other four parameters are inherent properties of the virus and remain constant. In addition, to account for reporting delays in reported cases [54], we introduce a Gaussian-distributed report delay term ($D_r$), which varies by month. Details of model inference and initialization are provided in Supplementary materials.

### Counterfactual simulations

We delve into different counterfactual scenarios to investigate the SSE of NPIs on regional COVID-19 trajectories further and to measure intervention effects by comparing cumulative confirmed cases in different scenarios. Due to the spatial dependence among regions, the NPIs in some specific key regions may exert larger SSE $\delta _{sp}$. Targeting these key regions and exploring the role of SSE in these specific regions on COVID-19 cases can help us maximize the impact of NPIs. To do this, we divide the US states into different groups based the intrastate human mobility, and implement different counterfactual scenarios within each group, specifically changing the intensity of different NPIs, determining the more effective regions, and restricting interstate human mobility.

Firstly, to locate appropriate intervention areas, we divide US states into four groups based on K-means clustering [55]. This algorithm, based on Euclidean distance, considers that the closer the distance between two targets, the greater the similarity. Therefore, we first define the distance between the targets. We calculate the average intensity of intrastate human mobility in each region from 6 January to 2 February. If the difference in human mobility within the two states is small, we assume that the two states are closer together. We then select the number of clusters based on the elbow method. Eventually, four different categories of regions are identified (Fig. 2A).

**Table 2:** The coefficients of different NPI estimates from the spatial econometric model

Phase	Phase A			Phase B		
Variables	Coef.	S.D	$95\%$ CI	Coef.	S.D	$95\%$ CI
Spatial effect	**0.6830**	0.0360	[0.6142, 0.7464]	**0.7790**	0.0280	[0.7241, 0.8329]
School closures	− 0.0002	0.0001	[− 0.0005, 0.0001]	**0.0004**	0.0001	[0.0003, 0.0006]
Workplace closures	**0.0005**	0.0001	[0.0003, 0.0007]	**0.0001**	0.0001	[0.0000, 0.0002]
Cancellation of public events	**0.0004**	0.0001	[0.0001, 0.0006]	**0.0001**	0.0000	[0.0001, 0.0002]
Restrictions on gatherings	**− 0.0002**	0.0001	[− 0.0004, − 0.0001]	**− 0.0001**	0.0000	[− 0.0002, − 0.0000]
Public transport closures	− 0.0000	0.0001	[− 0.0001,0.0001]	**− 0.0002**	0.0001	[− 0.0003, − 0.0001]
Stay-at-home orders	**0.0003**	0.0001	[0.0001, 0.0005]	**0.0002**	0.0001	[0.0001, 0.0003]
Restrictions on internal movements	0.0001	0.0001	[0.0000, 0.0003]	− 0.0001	0.0000	[− 0.0001, 0.0000]
Obs.	784			686		
$R^2$	0.9690			0.9740		

Our data includes 48 states (regions) and Washington D.C. Coef.: Posterior mean of coefficients. S.D.: Standard Deviation. We run a Markov chain of 50,000 iterations with a $50\%$ burn-in ratio. We treat the posterior mean of parameters as their Bayesian point estimates. We rely on the Bayesian $95\%$ CI to judge the significance of parameters. Bolded color indicates significance

## Results

### Estimation results of spatial econometric model

We first determine the spatial weight matrix by selecting the matrix with *K* maximum interaction (KMI, $K=8$) for Phases A and B based on the DIC described in Gelman [56] (See supplement materials). The flow matrix with the nearest *K* neighbors is a $49 \times 49$ interstate interaction matrix, with each region connected to its closest *K* neighbors. In this case, $K=8$, meaning each region has 8 neighbors that interact most frequently with it (Fig. 2B). Next, we employed the Bayesian MCMC method to estimate the panel SAR model. The estimation results are summarized in Table  2.

The findings reveal that the spatial effect is positive and significant in both Phase A and Phase B, indicating a strong interdependence among regions. This highlights the importance of considering spatial econometric models. When introducing NPIs, workplace closures, cancellation of public events, restrictions on gatherings, and stay-at-home orders show statistically significant coefficients. Except for restrictions on gatherings which would have a counteractive effect on reducing human mobility, the other three NPIs are found to have positive impacts. When lifting NPIs, all interventions are found to be statistically significant except for restrictions on internal movements. Notably, school closures had a more significant impact on reducing human mobility in Phase B as its coefficient is the largest among all NPIs.

### Direct and spatial spillover effects of NPIs on human mobility

Table 3 and Table 4 show the average direct and spatial spillover effects of each NPI estimated by Eq. 6. Most NPI impact estimates are significant, but not all NPIs can result in an increase in change of intrastate human mobility and thus a reduction in human mobility. Workplace closures, cancellation of public events, and stay-at-home orders have positive and significant direct effects and SSE that enlarge the changes in human mobility during both Phases A and B, whereas restrictions on gathering always exhibit negative and significant direct effects and SSE. During Phase A, an one standard-deviation increase in the intensity of workplace closures in one region can directly increase by 0.0006 in human mobility change in the region, which corresponds to 1,414 individuals in the mobility sample, while increasing the change of the human mobility in its neighboring region by 0.0010, corresponds to 2,356 individuals. Note that our mobile data is not the whole population, so the number of individuals represents only the number calculated based on our human mobility sample. But during Phase B, an one standard-deviation increase in the intensity of workplace closures in one region can only increase the change of the human movement in the region and its neighboring region by 0.0002 and 0.0004, respectively, corresponding to 372 and 745 individuals. During Phase A, workplace closures exert significant influences on restricting human movement. But during phase B, stay-at-home orders become more important. We also notice interventions such as restrictions on gathering and public transport closures tend to have negative impacts on the intrastate human mobility change. This may be because public transport closures may force people to resort to alternate modes of travel such as walking or driving, which may not effectively reduce human movement. Moreover, a strengthening of the restrictions on gatherings like indoor gatherings or dine-in may result in increased traffic as people tend to increase their outdoor communication instead. To demonstrate the robustness of the model findings, we also estimate the average direct effects and SSE of using the panel spatial Durbin model, and the results are similar to those estimated by the SAR model. For more information, see Supplementary materials for details.

**Table 3:** The coefficients estimates of average direct effects from spatial econometric model

Variables	Phase A			Phase B		
	Coef.	S.D.	$95\%$ CI	Coef.	S.D.	$95\%$ CI
School closures	$-$0.0002	0.0002	[$-$0.0005, 0.0001]	**0.0005**	0.0001	**[0.0003, 0.0007]**
Workplace closures	**0.0006**	0.0001	**[0.0004, 0.0008]**	**0.0002**	0.00005	**[0.0001, 0.0003]**
Cancellation of public events	**0.0004**	0.0001	**[0.0002, 0.0006]**	**0.0002**	0.00004	**[0.0001, 0.0003]**
Restrictions on gatherings	**− 0.0003**	0.0001	**[− 0.0005, − 0.0001]**	**− 0.0001**	0.00004	**[− 0.0003, − 0.0000]**
Public transport closures	$-$0.0000	0.0001	[$-$0.0002, 0.0001]	**− 0.0003**	0.0001	**[− 0.0004, − 0.0001]**
Stay-at-home orders	**0.0003**	0.0001	**[0.0001, 0.0005]**	**0.0002**	0.0001	**[0.0001, 0.0003]**
Restrictions on internal movements	**0.0002**	0.0001	**[0.0000, 0.0003]**	$-$0.0001	0.0001	[$-$0.0001, 0.0000]

Our data includes 48 states (regions) and Washington D.C. Coef.: Posterior mean of coefficients. S.D.: Standard Deviation. We run a Markov chain of 50,000 iterations with a $50\%$ burn-in ratio. We treat the posterior mean of parameters as their Bayesian point estimates. We also report the standard deviation of the posterior samples of parameters in parentheses. We rely on the Bayesian $95\%$ CI to judge the significance of parameters. Bolded color indicates significance

**Table 4:** The coefficients estimates of average spatial spillover effects from spatial econometric model

Variables	Phase A			Phase B		
	Coef.	S.D.	$95\%$ CI	Coef.	S.D.	$95\%$ CI
School closures	$-$0.0004	0.0003	[$-$0.0011, 0.0001]	**0.0020**	0.0003	**[0.0010, 0.0020]**
Workplace closures	**0.0010**	0.0002	**[0.0006, 0.0016]**	**0.0004**	0.0002	**[0.0000, 0.0010]**
Cancellation of public events	**0.0007**	0.0003	**[0.0003, 0.0013]**	** 0.0005**	0.0001	**[0.0002, 0.0009]**
Restrictions on gatherings	**-0.0005**	0.0001	**[− 0.0009, − 0.0002]**	**− 0.0004**	0.0001	**[− 0.0006, − 0.0001]**
Public transport closures	$-$0.0000	0.0001	[$-$0.0003, 0.0002]	**− 0.0008**	0.0002	**[− 0.0010,− 0.0003]**
Stay-at-home orders	**0.0006**	0.0002	**[0.0002, 0.0011]**	**0.0006**	0.0002	**[0.0002, 0.0011]**
Restrictions on internal movements	**0.0003**	0.0001	**[0.0000, 0.0006]**	$-$0.0001	0.0001	[$-$0.0005, 0.0001]

Our data includes 48 states (regions) and Washington D.C. Coef.: Posterior mean of coefficients. S.D.: Standard Deviation. We run a Markov chain of 50,000 iterations with a 50$\%$ burn-in ratio. We treat the posterior mean of parameters as their Bayesian point estimates. We also report the standard deviation of the posterior samples of parameters in parentheses. We rely on the Bayesian 95$\%$ CI to judge the significance of parameters. Bolded color indicates significance

**Fig. 3:**
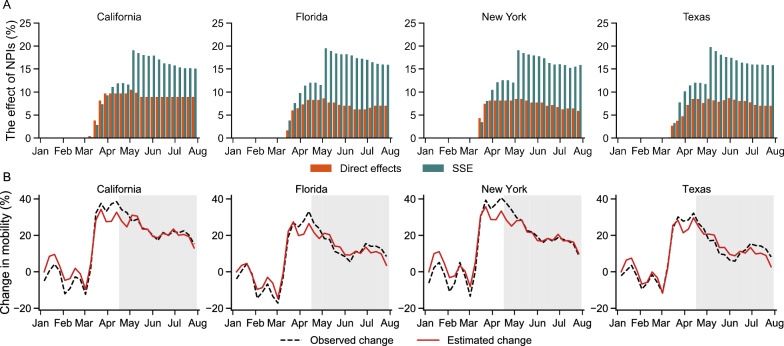
The effects of all NPIs estimated by the panel SAR model. **A** The direct effects and SSE of all NPIs in four representative regions: California, Florida, New York, and Texas are shown. **B** Comparison of the change of human movement estimated by the spatial econometric model versus factual value. The white (shaded) area indicates the introduction (lifting) phase in which interventions were initialized (relaxed). Results for all regions are presented in Supplementary materials

In addition to the average direct effects and SSE of the NPIs, we also calculate and isolate the direct effects and SSE of all NPIs according to Eq. 7. The results indicate that the SSE of all NPIs on human mobility during Phase A and Phase B turns out to be stronger than direct effects (Fig. 3A). This demonstrates that NPIs in neighboring regions can significantly influence human mobility in one region. Then, we can combine the overall direct effects and SSE of all interventions, estimate curves of change in intrastate human mobility for regions, and compare them with factual observed mobility (Fig. 3B). The observed and estimated curves are fairly close for most regions, with results for all regions presented in Supplementary materials. And the $R^2s$ during phases A and B are 0.969 and 0.974, respectively, indicating that our model can accurately describe changes in human mobility.

### S-SEIR model for disease transmission

**Fig. 4:**
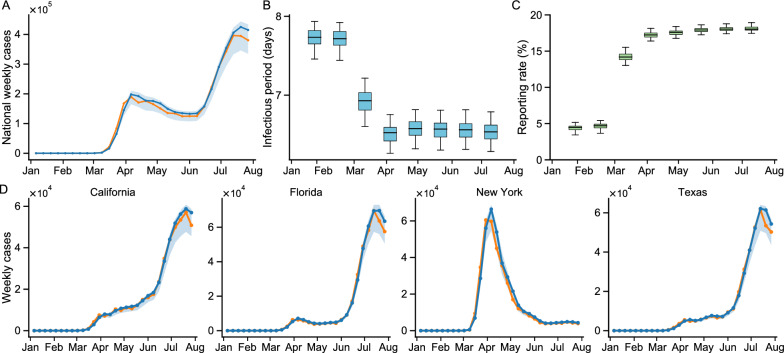
Model fitting and parameter inference of the S-SEIR model. **A** Model fitting to weekly case numbers (orange line) in the United States. The line and shaded area represent the median and $95\%$ CI, respectively. The blue line means the estimated cases and the orange line means the reported cases. **B** Distribution of infectious periods in the United States. **C** Distribution of report rates in the United States. Monthly posterior estimates from January 6 to August 2, 2020, are provided. In **B** and **C**, the center line shows the median, box bounds represent 25*th* ($Q_1$) and 75*th* ($Q_3$) percentiles, and whiskers show $Q_1-1.5 \times IQR$ and $Q_3 + 1.5 \times IQR$. IQR means interquartile range. **D** Model fitting to weekly case numbers in the four states (regions) with the most cases. All distributions in this figure are from $n = 100$ ensemble members

By incorporating the estimated changes in human mobility within states into the S-SEIR model, we evaluate the role of SSE of NPIs in COVID-19 transmission. We calibrate the model for each region by minimizing the RMSE to the weekly confirmed case. The RMSE on weekly confirmed cases is 500, and the Pearson *R* is 0.98. The fitted model can capture the trajectory of weekly confirmed cases well at the national (Fig. 4A) and state-level (Fig. 4D). Results for each individual region are presented in Supplementary materials. The model structure and calibration also allow us to estimate the infectious period and reporting rate at the national and state levels, with the national-level results displayed in Fig. 4. The national infectious period (*D*) decreased from 7.72 days ($95\%$ CI: 7.50$-$7.91) during January and February 2020 to 6.50 days ($95\%$ CI: 6.26$-$6.97) during March and August 2020. Additionally, we find that the national reporting rate ($\mu$) is low but gradually increased over time. In particular, $\mu$ is only about $4.53\%$ ($95\%$ CI: 3.70 - $5.14\%$) from January to February 2020. Many early missed diagnoses may also be responsible for the rapid increase in infections. These inference results are robust to different parameter settings and model configurations.

**Fig. 5:**
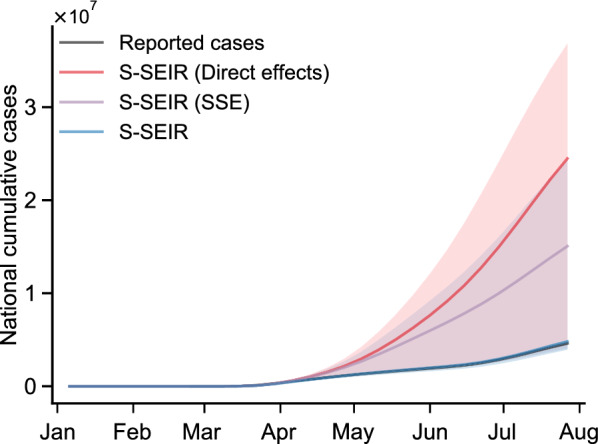
The contribution of direct effects and SSE of all NPIs on national cumulative cases. The line and shaded area represent the median and $95\%$ CI, respectively

Based on the S-SEIR model, we can assess the impact of both the direct effects and the SSE of all NPIs on COVID-19 transmission. To compare the effect of SSE over the entire study period (Fig. 5), we quantify the contribution of direct effects and SSE using cumulative confirmed cases as an indicator:

12 $$\begin{aligned} C_{direct}= & {} \frac{Case_{no} - Case_{direct}}{Case_{no} - Case_{S-SEIR}} \end{aligned}$$

13 $$\begin{aligned} C_{spillover}= & {} \frac{Case_{no} - Case_{spillover}}{Case_{no} - Case_{S-SEIR}} \end{aligned}$$

where $C_{direct}$ and $C_{spillover}$ denote the contribution of the direct effects and SSE of NPIs to COVID-19 cases, respectively. $Case_{no}$ is the cumulative cases estimated by no change in human mobility, $Case_{direct}$ is the cumulative cases estimated by only direct effects of NPIs. $Case_{spillover}$ is the cumulative cases estimated by only spillover effects of NPIs. $Case_{S-SEIR}$ is the cumulative cases estimated by S-SEIR model. $Case_{no} - Case_{S-SEIR}$ corresponds to the number of cases that can be reduced by the change of intrastate human mobility. In all the scenarios mentioned above, we assume interregional human flows keep consistent, and only focus on the contribution of direct effects and SSE of NPIs within regions calculated by S-SEIR model.

We find that the SSE of all NPIs can explain the $61.2\%$ ($95\%$ CI: 52.8-$84.4\%$) of national cumulative confirmed cases, while the direct effects of NPIs can explain the $27.2\%$ ($95\%$ CI: 21.3-$45.2\%$). The results suggest that the NPIs of one’s neighbors exhibit a larger influence on the transmission of COVID-19 in one region. This may be due to the fact that, in the early stages of the pandemic, regions with fewer locally reported cases place greater emphasis on preventing the spread of the virus from neighboring regions. As a result, more attention is paid to the interventions and confirmed cases in neighboring regions.

### Comparing the effectiveness of region-based interventions

Our objective is to compare the effectiveness of different region-based intervention strategies. We also aim to examine the impact of SSE in these regions and determine if it is linked to population size or intrastate human mobility. We divide the regions into four groups based on the intrastate human mobility and K-means clustering method. The four groups are (1) Region 1 ($r_1$): 22 regions with relatively low human mobility (e.g., Nevada, Montana); (2) Region 2 ($r_2$): 18 regions with intermediate human mobility (e.g., Wisconsin, Washington); (3) Region 3 ($r_3$): 6 regions with higher human mobility (e.g.New York, Illinois); (4) Region 4 ($r_4$): 3 regions with the highest human mobility. In addition, we define Region 5 ($r_5$) including the $r_3$ and $r_4$. All subsequent analyses will be based on these five intervention regions.

**Fig. 6:**
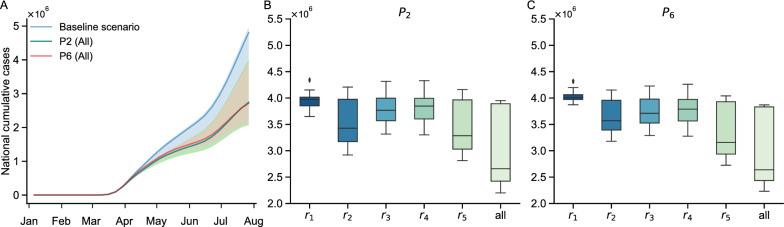
The estimated counterfactual national cumulative confirmed cases by strengthening NPIs.**A** The estimated counterfactual national cumulative confirmed cases by strengthening the intensity of $P_2$ and $P_6$ in all regions. The intensity of each NPI is set to 100. The baseline scenario is the epidemic curve estimated by the S-SEIR model. **B**–**C** The counterfactual national cumulative confirmed cases at $t = 30$ week. The intensity of each NPI is set to 100. **B** strengthening the intensity of $P_2$ in different intervention regions. **C** Strengthening the intensity of $P_6$ in different intervention regions. In **B**– **C**, the center line shows the median, box bounds represent 25*th* ($Q_1$) and 75*th* ($Q_3$) percentiles, and whiskers show $Q_1-1.5 \times IQR$ and $Q_3 + 1.5 \times IQR$. IQR means interquartile range. All distributions are obtained from $n = 100$ ensemble members

We first altered the intensities of specific NPIs in the targeted regions, while keeping others constant. We evaluate the impact of this targeted intervention by comparing the counterfactual cumulative cases at the national level with actual cases (Fig. 6). Our spatial econometric model aids us in determining the NPIs that are effective in reducing intrastate human mobility. We anticipate that these same NPIs may also have a substantial impact on reducing COVID-19 cases. To test this, we selected the NPIs that had the greatest impact on reducing human mobility, namely workplace closures ($P_2$) and stay-at-home orders ($P_6$), increased their intensities to 100, and incorporated these enhanced NPIs into the S-SEIR model for each region group to generate the counterfactual cases.

We observe that the implementation of enhanced $P_2$ and $P_6$ in different regions leads to a comparable reduction in the national number of confirmed cases. We find that the regions $r_5$ and $r_2$ are the more effective targets for interventions, especially when not intervening in all forty-nine regions simultaneously. Importantly, when fully implemented in $r_2$ and $r_5$, a 100 intensity $P_2$ intervention results in a comparable reduction in the number of national confirmed cases for both scenarios. The reduction in cases is estimated to be 2,177,620 ($95\%$ CI: 113,630–2,692,363) when implemented in $r_2$ and 2,198,963 ($95\%$ CI: 165,130–2,664,901) when implemented in $r_5$. A concentrated intervention with $P_6$ in $r_5$ yields even greater reduction in confirmed cases compared to $r_2$. The reduction in cases is estimated to be $26.1\%$ ($95\%$ CI: 1.5-$34.2\%$) with $P_6$ in $r_2$, corresponding to 1,261,481 ($95\%$ CI: 58,873–1,688,624) cases. Meanwhile, The reduction in cases is estimated to be $34.7\%$ ($95\%$ CI: 2.1-$43.7\%$) within $P_6$ in $r_5$, corresponding to 1,676,151 ($95\%$ CI: 82,696–2,159,600) cases.

Additionally, the intervention in $r_5$ proves to be a close second to intervention in all forty-nine regions. If a targeted intervention on nine regions in $r_5$ with 100 intensity $P_2$ and $P_6$, the number of cases compared to intervening in all regions would increase by only $23.1\%$ ($95\%$ CI: 1.6-$28.5\%$) and $19.4\%$ ($95\%$ CI: 2.1-$22.8\%$) respectively. Our findings suggest that strengthening NPIs in regions with high human mobility can have a significant impact in reducing the number of COVID-19 cases nationwide. Particularly, the NPIs in $r_5$ exhibit a greater spillover effect compared to other groups, and therefore, more intensive interventions for the nine high human mobility regions in $r_5$ may have a considerable effect in reducing cases across the country.

### Restricting the interstate human mobility

The effectiveness of lockdown policies in flattening the COVID-19 epidemic curve has been well-established in previous studies [57, 58]. Here we investigate various targeted lockdown scenarios, from a complete lockdown strategy where all movements in and out of targeted regions are prohibited, to a partial lockdown strategy that restricts either origin-based or destination-based flows. The superiority of a complete lockdown over a partial lockdown strategy is yet to be determined. Moreover, we are also not sure whether a lockdown of regions with higher human mobility can result in greater reduction in COVID-19 cases. Hence, we generate counterfactual simulations for complete or partial lockdown on different regional groups to answer those questions.

**Fig. 7:**
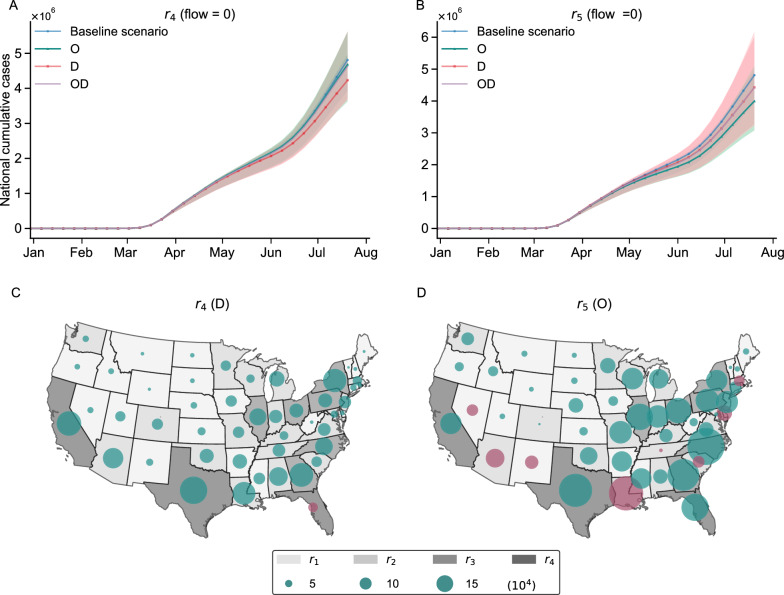
The estimated counterfactual national cumulative confirmed cases by lockdown in $r_4$ **or** $r_5$ **.** We consider the baseline scenario as the epidemic curve estimated by S-SEIR model for **A**, **B**, **C**, **D**. **A** The estimated counterfactual national cumulative confirmed cases by lockdown in $r_4$, which means the human mobility between some regions and $r_4$ is set to 0, including travel flow from $r_4$ (denoted as ’O’) and travel flow to $r_4$ (denoted as ’D’). (B) The estimated counterfactual national cumulative confirmed cases by lockdown the flow in $r_5$. (C) The median differences in estimated national cumulative confirmed cases at $t = 30$ week by the destination-based lockdown in $r_4$ and baseline scenario. (D) The median differences in estimated national cumulative confirmed cases at $t = 30$ week by the destination-based lockdown in $r_5$ and baseline scenarios. The size of the circle indicates the absolute value of the differences. Bottle green indicates that the cases estimated by counterfactual scenarios are fewer than the baseline. Purple indicates the cases estimated by counterfactual scenarios are higher. All distributions are obtained from $n = 100$ ensemble members

Our findings suggest that the complete lockdown strategy does not seem to be more effective than the partial lockdown strategy (Fig. 7A, B). Taking $r_4$ and $r_5$ as examples, when $r_4$ is targeted, the destination-based lockdown is the most effective one that gives the lowest number of cases. On the other hand, when $r_5$ is targeted, the origin-based lockdown becomes the most effective strategy. Additionally, we observe that lockdown of more regions with higher human mobility can deliver fewer confirmed cases nationwide. Particularly, a targeted lockdown on $r_5$ can decrease more cases than targeting at $r_4$. When the lockdown (whether complete or partial lockdown) is given on $r_5$, there is a maximum reduction of 587,194 (95 $\%\hbox {CI}$: 69,206–976,481) cumulative nationwide confirmed cases, while given on $r_4$, there is a maximum reduction of 47,920 (95 $\%\hbox {CI}$: -356,401–123,269) cases. But this observation should be interpreted with caution and the decrease in cases can not be simply attributed to the increase in the number of targeted regions. See Supplementary materials for more discussion.

Although from a national perspective, lockdown-type policies manage to reduce cumulative confirmed cases. But not every region obtains an equally significant reduction in the number of cases. Rather, the difference from the baseline scenario exhibits obvious spatial heterogeneity (Fig. 7C, D). Targeted lockdown on $r_4$ could significantly reduce cases in almost all regions, with the exception of Florida having a slight increase in cases. For $r_5$, an origin-based partial lockdown is able to lower cases in most regions, especially North Carolina. But the cases in some regions may increase slightly, particularly in Louisiana and some of the neighboring regions of California. This is because North Carolina closely interacts with most regions in $r_5$. Origin-based lockdown of $r_5$ reduces the importation risk into North Carolina, thus lowering the number of confirmed cases. In contrast, Louisiana only has strong interactions with Texas and relatively infrequent interactions with other regions of $r_5$. Origin-based lockdown of $r_5$, although reduces the importation risk from Texas to Louisiana, also affects the exportation of Louisiana to Texas, thus leading to an increase in the susceptible population and the local transmission risk in Louisiana within the S-SEIR system. In general, we do not recommend a complete lockdown strategy for targeted regions and instead opting for either origin or destination-based partial lockdown, which seems to be more effective. The influence of lockdown-type policies may also vary depending on the targeted region group, so the choice of which OD flows to restrict should be based on the specific region being intervened.

## Discussion and conclusions

In this work, we quantify the spillover effects of different NPIs on human mobility and pandemic risk and find that 1) the spatial spillover effects of NPIs can explain $61.2\%$ [$95\%$ CI: 52.8-$84.4\%$] of national cumulative confirmed cases, This suggests that the NPI spillovers exhibit significant impact on COVID-19 transmission across regions, and the presence of the spillover effect significantly enhances the NPI influence. 2) strengthening NPIs in the key regions with high internal human mobility can significantly reduce the cumulative case nationwide. Local governments can take into account possible SSE of NPIs from neighboring regions when adjusting their own policies. Thus, our findings call for collaboration and resource coordination across countries and regions to combat the pandemic [59–61].

Strong NPI spillovers imply that regional epidemics may be heavily affected by interventions from neighboring regions. Even if certain regions do not implement strict NPIs, their epidemic curve can be effectively flattened by the SSE of NPIs in neighboring regions. This phenomenon also occurs in vaccination, where the spillover effects of vaccination have been extensively studied in the literature [62, 63], with herd immunity being a good example. Few literatures has discussed the spillover effects of different NPIs as we do. Examination of the spillover effects of NPIs and vaccines is equally important and can be combined in the future for better regional resource coordination [64]. In addition, the NPIs in these key regions with frequent human mobility have greater spillover effects and reduce more cases. This may be due to the fact that these regions usually interact more frequently with other regions, similar to a hub node in a complex network. However, these regions may also be more vulnerable, and significantly strengthening NPIs in these regions may result in greater economic loss and investment. Although this makes coordination more difficult in the real world, our simulation provides a more moderate scenario (see Supplementary materials). Region-based interventions are still effective even when NPIs are strengthened at very low intensities.

This study also has some limitations. First, the dataset we use does not cover all people, and there may be a bias in extracting interstate interactions. We propose a movement adjustment factor ($\theta$), but it may not reflect real-world human movement accurately. Although our S-SEIR model manages to assess the relationship of NPIs’ SSE, human mobility, and virus transmission, mobility data that represent a larger proportion of the population are still necessary. Secondly, we use the reduction in human mobility to completely replace the removed susceptible contacts in the disease transmission. In addition to social distancing, the decrease in susceptible contacts may also be related to contact tracing, isolation, etc. Further exploration of the more complex mechanisms of human mobility variations will lead to a deeper understanding of the policy effects.

Our study quantifies the spillover effects across regions and clarifies the intervention range, and provides an operational insight for how to search for the most effective region-based policies conditional on spillovers. This finding will not only contain the current outbreak, but also help us determine better response strategies when dealing with similar challenges, such as influenza, monkeypox, dengue fever, and respiratory syncytial virus. Moreover, our modeling framework may also be modified or generalized to other contexts such as crime [19] and traffic [65] where meaningful spillover effects are present and collaborations among different regions are important.

## Supplementary Information

**Additional file 1: Table S1. **Best-fit model posterior estimates of key epidemiological parameters for January 6 to March 9, 2020. **Table S2. **Gamma distributions for monthly reporting delay (Dr). **Table S3.** Comparison of the results of DIC based on different spatial weight matrices. **Table S4.** The contribution of direct effects and SSE of all NPIs on COVID-19 cases in 48 regions and Washington D.C. **Table S5.** The coefficients of different NPI estimates under the dependent variable multiplied by 100. **Table S6.** The coefficients estimate of direct effects under the dependent variable multiplied by 100. **Table S7.** The coefficients estimate of spatial spillover effects under the dependent variable multiplied by 100. **Table S8.** The coefficients estimate of direct effects from spatial panel durbin model. **Table S9.** The coefficients estimate of spatial spillover effects from spatial panel durbin model. **Table S10.** The coefficient estimated by our spatial panel model with different weight matrix during Phase A. **Table S11.** The coefficient estimated by our spatial panel model with different weight matrix during Phase B. **Figure S1.** The connectivity structure of different spatial weight matrix. **Figure S2.** Comparison of the change of human mobility estimated by our spatial panel model versus the factual human mobility change of 48 states and Washington D.C. **Figure S3.** The scatterplot of  the change of human mobility estimated by our spatial panel model versus the factual human mobility change in all states. **Figure S4.** The direct and spatial spillover effect estimates of NPIs in 48 states and Washington D.C. from the spatial panel model. **Figure S5.** Model fitting to daily case numbers (blue dots) by S-SEIR model in 48 states and Washington D.C. ** Figure S6.** The estimated cases by different model in 48 states (regions) and Washington D.C. Notes: S-SEIR (Direct effects): similar to the S-SEIR model, but only the direct effects of NPIs are considered. S-SEIR (SSE): similar to the S-SEIR model, but only the spatial spillover effects of NPIs are considered. S-SEIR: both the direct and spatial spillover effects are considered. The line and shaded area represent the median and 95% CI, respectively. ** Figure S7.** The estimated national cumulative confirmed cases by changing the intensity of all NPIs in different regions. **Figure S8.** The national cumulative confirmed cases estimated by changing the intensity of diffferent NPIs in different regions. **Figure S9.** The estimated national cumulative confirmed cases by restricting the flows of r2, r4, or r5, including all travel flow with these targeted regions (denoted as ’OD’), travel flow from these targeted regions (denoted as ’O’) and travel flow to these targeted regions (denoted as ‘D’). **Figure S10.** The median differences in national cumulative confirmed cases at t = 30 week are estimated by the complete lockdown (OD), origin-based lockdown (O), destination-based lockdown (D) in r4 or r5 and baseline scenario. **Figure S11.** The change of intrastate human mobility in each state. ** Figure S12.** The distribution of the dependent variables in different weeks. **Figure S13.** Estimation results of the change of intrastate human mobility and spatial spillover effects of NPIs by panel SAR model. The matrix during introduction phase is flow weight (8 neighbors) matrix, during lifting phase is the inverse distance matrix with a cut-off distance of 600 km.  **Figure S14.**  Estimation results of the change of intrastate human mobility and spatial spillover effects of NPIs by panel SAR model. The matrix during introduction phase is the inverse distance matrix with a cut-off distance of 600 km, and during lifting phase is flow weight (8 neighbors). ** Figure S15.** Estimation results of the change of intrastate human mobility and spatial spillover effects of NPIs by panel SAR model. The matrix during introduction phase is the inverse distance matrix with a cut-off distance of 600 km, and during lifting phase is the inverse distance (the max distance is 800 km). **Figure S16.** Estimation results from the S-SEIR model, taking changes in actual human mobility as intervention effects. **Figure S17.** Estimation results from the Sp-SEIR model, the initial seed is the number of cases in the five days from T0 to T0+4. **Figure S18.** Estimation results from the Sp-SEIR model. During initialization, symptomatic patients are not assigned an initial value. **Figure S19.** Estimation results from the Sp-SEIR model, the range of movement fixed factor (θ) is from 1 to 10. **Figure S20.** Estimation results from the Sp-SEIR model. We assume that the infection of asymptomatic patients relative to symptomatic patients also varies over time and region.

## Data Availability

The state-level COVID-19 case data in the US are available at Johns Hopkins University coronavirus resource center (http://github.com/CSSEGISandData/COVID-19/tree/master/csse_covid_19_data/csse_covid_19_time_series). Human mobility dataset is provided by Kang et al. (https://github.com/GeoDS/COVID19USFlows). The policy data are downloaded from The Oxford COVID-19 Government Response Tracker (OxCGRT) (https://github.com/OxCGRT/covid-policy-tracker). The codes will be available on GitHub, at https://github.com/giswkl/S-SEIR.
