# Supplementary material for: Quantifying the spatial spillover effects of non-pharmaceutical interventions on pandemic risk
Source: Int J Health Geogr. 2023 Jun 7;22:13. doi: 10.1186/s12942-023-00335-6 (PMC10245369; doi:10.1186/s12942-023-00335-6)
Supplement: Supplementary file 1 — Additional file 1: Table S1. Best-fit model posterior estimates of key epidemiological parameters for January 6 to March 9, 2020. Table S2. Gamma distributions for monthly reporting delay (Dr). Table S3. Comparison of the results of DIC based on different spatial weight matrices. Table S4. The contribution of direct effects and SSE of all NPIs on COVID-19 cases in 48 regions and Washington D.C. Table S5. The coefficients of different NPI estimates under the dependent variable multiplied by 100. Table S6. The coefficients estimate of direct effects under the dependent variable multiplied by 100. Table S7. The coefficients estimate of spatial spillover effects under the dependent variable multiplied by 100. Table S8. The coefficients estimate of direct effects from spatial panel durbin model. Table S9. The coefficients estimate of spatial spillover effects from spatial panel durbin model. Table S10. The coefficient estimated by our spatial panel model with different weight matrix during Phase A. Table S11. The coefficient estimated by our spatial panel model with different weight matrix during Phase B. Figure S1. The connectivity structure of different spatial weight matrix. Figure S2. Comparison of the change of human mobility estimated by our spatial panel model versus the factual human mobility change of 48 states and Washington D.C. Figure S3. The scatterplot of the change of human mobility estimated by our spatial panel model versus the factual human mobility change in all states. Figure S4. The direct and spatial spillover effect estimates of NPIs in 48 states and Washington D.C. from the spatial panel model. Figure S5. Model fitting to daily case numbers (blue dots) by S-SEIR model in 48 states and Washington D.C. Figure S6. The estimated cases by different model in 48 states (regions) and Washington D.C. Notes: S-SEIR (Direct effects): similar to the S-SEIR model, but only the direct effects of NPIs are considered. S-SEIR (SSE): similar to the S-SEIR model, bu [file 12942_2023_335_MOESM1_ESM.pdf]

Supplementary materials for Quantifying the spatial spillover effects of non-pharmaceutical interventions on pandemic risk

Keli Wang[1,2], Xiaoyi Han[3,4], Lei Dong[1,2],Xiao-Jian Chen[1,2],

Gezhi Xiu[1,2],Mei-Po Kwan[5,6] and Yu Liu[1,2,*]

June 4, 2023

Methods

Overview of Bayesian estimation of panel spatial autoregressive model

We use the Bayesian Markov Chain Monte Carlo (MCMC) method to estimate the panel spatial autoregressive (SAR) model in the main text during the introduction and lifting phases separately. Let $\Theta = (\rho, \boldsymbol{\lambda}, \sigma^2)$ be the vector of common parameters of SAR model. Also let $E(\rho) = I_n - \rho \boldsymbol{W}$ and $H_t(\Theta, \boldsymbol{\nu}, \xi_t) = E(\rho)\boldsymbol{Y_t} - \boldsymbol{X_{t-1}}\boldsymbol{\lambda} - \boldsymbol{\nu} - l_N \xi_t$. The likelihood function of the model at time t is

$$f(\boldsymbol{Y}_t|\Theta, \boldsymbol{\nu}, \xi_t) \propto (\sigma^2)^{-\frac{n}{2}} \times |E(\rho)| \times \exp(-\frac{H_t(\Theta, \boldsymbol{\nu}, \xi_t)' H_t(\Theta, \boldsymbol{\nu}, \xi_t)}{2\sigma^2}). \tag{S1}$$

For simplicity, the pre-determined explanatory variables $\{\boldsymbol{X}_{t-1}\}_{t=1}^T$, the regional and time effects $\boldsymbol{\nu}$ and $\{\xi_t\}_{t=1}^T$, as well as the exogenous spatial weights $\boldsymbol{W}$ are suppressed from the conditional set of the likelihood function. Notice that we need to impose some stability conditions on $\boldsymbol{\rho}$ to ensure $E(\boldsymbol{\rho})$ is invertible so the reduced form of (SAR model) exists. As we specify a row-normalized $\boldsymbol{W}$ with $\|\boldsymbol{W}\|_\infty$, we follow Lee and Liu [1] to consider the following stability condition which is computationally simple:

$$\|\rho \boldsymbol{W}\|_\infty \leq |\rho| \times \|\boldsymbol{W}\|_\infty = |\rho| < 1. \tag{S2}$$

This condition would be imposed on the MCMC sampling step for the spatial parameter ρ. Assume the following priors for Θ:

$$\rho \sim \mathcal{U}(-1,\ 1),\ \boldsymbol{\lambda} \sim \mathcal{N}(\lambda_0, \Sigma_{\lambda_0}),\ \sigma^2 \sim \mathcal{IG}(\frac{a}{2}, \frac{b}{2}),$$
$$\boldsymbol{\nu} \sim \mathcal{N}(\nu_0, \Sigma_{\nu_0}),\ \xi_t \sim \mathcal{N}(\xi_0, \sigma_\xi^2),\ t = 2, 3, \cdots, T. \tag{S3}$$

We mostly follow LeSage and Pace [2] and Han et al. [3] to specify the priors for Θ, such as the uniform prior for ρ, the normal prior for $\boldsymbol{\lambda}$, $\boldsymbol{\nu}$ and ξ_t's, and the inverse gamma prior

for σ^2. Particularly, we set the upper and lower bound of ρ to be, respectively 1 and -1 for convenience, which would automatically guarantee (S2) holds. In our empirical studies, we set $\lambda_0 = 0$, $\Sigma_\lambda = 1000 I_7$, $a = 0.001$, $b = 0.001$, $\nu_0 = 0$, $\Sigma_\nu = 10 I_{49}$, $\xi_0 = 0$, and $\sigma_\xi^2 = 1$. Those hyperparameter settings generally lead to relatively uninformative prior distributions over the parameter spaces.

With the prior distribution $\pi(.)$ in (S3), the posterior distribution of all parameters is

$$p(\Theta, \boldsymbol{\nu}, \{\xi_t\} | \{\boldsymbol{Y}_t\}) \propto \pi(\rho) \times \pi(\boldsymbol{\lambda}) \times \pi(\boldsymbol{\nu}) \times \pi(\{\xi_t\}) \times f(\boldsymbol{Y}_t | \Theta). \tag{S4}$$

With conjugate priors, $\boldsymbol{\lambda}$, σ^2, $\boldsymbol{\nu}$ and ξ_t's can be directly simulated from their posterior distributions, which is either normal or inverse-gamma distributions, via Gibbs sampling steps. But a Metropolis-Hasting (M-H) step is needed to obtain the posterior sample of ρ because the corresponding posterior distribution does not take a known form. The whole MCMC sampler can be realized through the following steps:

- Step 1: Sample ρ from $p(\rho | \{\boldsymbol{Y}_t\}, \boldsymbol{\lambda}, \sigma^2, \boldsymbol{\nu}, \{\xi_t\})$ using a M-H step;
- Step 2: Sample $\boldsymbol{\lambda}$ from $p(\boldsymbol{\lambda} | \{\boldsymbol{Y}_t\}, \rho, \sigma^2, \boldsymbol{\nu}, \{\xi_t\})$ using a Gibbs step;
- Step 3: Sample σ^2 from $p(\sigma^2 | \{\boldsymbol{Y}_t\}, \rho, \boldsymbol{\lambda}, \boldsymbol{\nu}, \{\xi_t\})$ using a Gibbs step;
- Step 4: Sample $\boldsymbol{\nu}$ from $p(\boldsymbol{\nu} | \{\boldsymbol{Y}_t\}, \rho, \boldsymbol{\lambda}, \sigma^2, \{\xi_t\})$ using a Gibbs step;
- Step 5: Sample ξ_t from $p(\xi_t | \boldsymbol{Y}_t, \rho, \boldsymbol{\lambda}, \sigma^2)$ using a Gibbs step for $t = 2, 3, \cdots, T$.

The length of the MCMC sampler is 50000 iterations with a 50% burn-in ratio. We treat the posterior mean of MCMC samples as point estimates for parameters and use the Bayesian 95% credible interval (CI) to judge the significance of parameter estimates.

The spatial weights matrix

The spatial weight matrix $\boldsymbol{W} = [W_{ij}]$ reflects the degree of adjacency or association between spatial units that can usually be constructed based on the adjacency or distance relationship [4]. We first choose an inverse distance spatial weight matrix and set truncation d_{max} to select neighbors closer to each region. The states beyond a specific distance d_{max} are regarded as unconnected ($W_{ij} = 0$).

However, infectious diseases can spread between two geographically distant regions, because geographical proximity does not fully capture air-traffic-mediated epidemics [5]. Thus, determining neighbors merely from geographical distance is not sufficient. To overcome this limitation, we use the spatial interaction distance, which characterizes the dependence relationship by human interaction across states. Following the concept of K nearest neighbors (KNN), we construct the K maximum interaction matrix (KMI) based on the spatial interaction distance. Like KNN, the most important part of the KMI method is the choice of K-value and the distance calculation. We define the distance by OD flows between region i and j as: $d(i,j) = flow_{ij}$, if $i = j$, $d(i,i) = 0$. Then we sort the distance of region i from the other regions in descending order. We assume the first k regions with larger distances are connected to region i, and the others are viewed as unconnected. Therefore, the weight between region i and j is: $W_{ij} = d_k(i,j)$. If $d_k(i,j)$ does not belong to the first k distances, then $d_k(i,j) = 0$, otherwise $d_k(i,j) = d(i,j)$. The KMI is chosen by the state-to-state interaction from 6 January to 2 February 2020 to ensure the stability of neighbors.

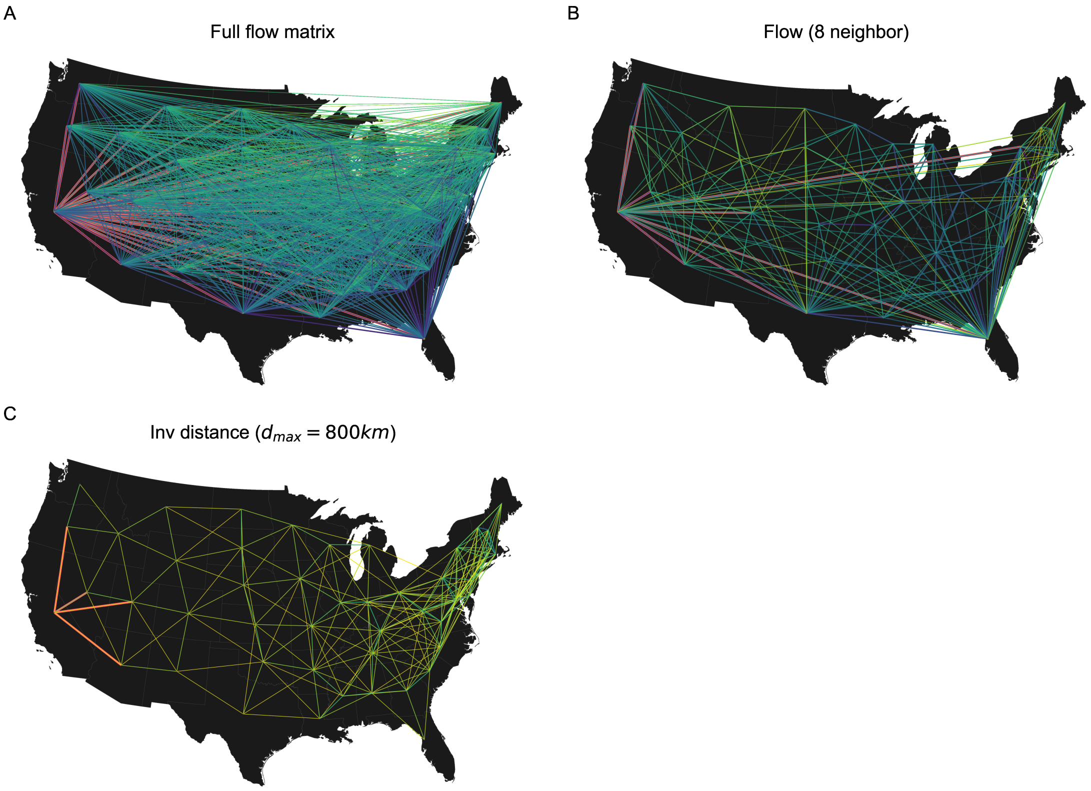

Figure S1: The connectivity structure of different spatial weight matrix. (A) full flow matrix: 49×49 full matrix of interstate interactions. (B) flow matrix with nearest 8 neighbors: 49×49 interstate interaction matrix, where each region is only connected to its closest 8 neighbors. (C) Inv distance matrix with a cut-off distance ($d_{max} = 800$ km): 49×49 inverse distance matrix with a cut-off distance of 800 km, otherwise 0. (D) 49×49 inv distance matrix with a cut-off distance ($d_{max} = 600$ km). In each subfigure, purple or orange lines represent neighbors of California. The color indicates the size of element of the spatial weights. The darker the color, the larger the weight value.

Selection of spatial weights matrices from DIC

We apply the deviation information criterion (DIC), proposed by Spiegelhalter et al. [6], to determine which spatial weight matrix is more suitable for the model. DIC can be viewed as the Bayesian version of Akaike information criterion (AIC) in Akaike [7]. Similar to AIC, DIC trades off a measure of model fit against a measure of complexity. As with AIC, the smaller DIC is, the better the model is. Let $\mathbf{Q}$ denote the parameter of the spatial model (including the regional and time effects). Here we adopt the DIC in Gelman.et al [8], namely,

$$DIC = \bar{D} + P_{DV}. \tag{S5}$$

Specifically, $\bar{D} = E_{\mathbf{Q}|\{\mathbf{Y}_t\}}(-2lnf(\{\mathbf{Y}_t\}|\mathbf{Q})$ is defined as the Bayesian measure of model fit, which is essentially equal to minus 2 times the posterior mean of the likelihood function. On the other hand, the second term $P_{DV} = 2 \times Var_{\mathbf{Q}|\{\mathbf{Y}_t\}} lnf(\{\mathbf{Y}_t\}|\mathbf{Q})$, which is two times the variance of the posterior samples of the log likelihood function, captures the effective number of parameters in the model. We evaluate the value of (S5) for different choices of $\boldsymbol{W}$ using the posterior draws of parameters, and select the $\boldsymbol{W}$ that gives the smallest DIC.
